# Positive geotactic behaviors induced by geomagnetic field in *Drosophila*

**DOI:** 10.1186/s13041-016-0235-1

**Published:** 2016-05-18

**Authors:** Ji-Eun Bae, Sunhoe Bang, Soohong Min, Sang-Hyup Lee, Soon-Hwan Kwon, Youngseok Lee, Yong-Ho Lee, Jongkyeong Chung, Kwon-Seok Chae

**Affiliations:** Department of Biology Education, Kyungpook National University, 80 Daehakro, Bukgu, Daegu, 41566 Korea; Department of Nanoscience & Nanotechnology, Kyungpook National University, Daegu, Korea; National Creative Research Initiatives Center for Energy Homeostasis Regulation, Institute of Molecular Biology and Genetics and School of Biological Sciences, Seoul National University, Seoul, 151-742 Korea; Department of Bio and Fermentation Convergence Technology, Kookmin University, Seoul, Korea; Brain and Cognition Measurement Laboratory, Korea Research Institute of Standards and Science, Daejeon, Korea; Brain Science and Engineering Institute, Kyungpook National University, Daegu, Korea

**Keywords:** Cryptochrome, *Drosophila melanogaster*, Geomagnetic field, Gravity, Johnston’s organ, Negative geotaxis, Positive geotaxis, Vertical movement

## Abstract

**Background:**

Appropriate vertical movement is critical for the survival of flying animals. Although negative geotaxis (moving away from Earth) driven by gravity has been extensively studied, much less is understood concerning a static regulatory mechanism for inducing positive geotaxis (moving toward Earth).

**Results:**

Using *Drosophila melanogaster* as a model organism, we showed that geomagnetic field (GMF) induces positive geotaxis and antagonizes negative gravitaxis. Remarkably, GMF acts as a sensory cue for an appetite-driven associative learning behavior through the GMF-induced positive geotaxis. This GMF-induced positive geotaxis requires the three geotaxis genes, such as *cry*, *pyx* and *pdf*, and the corresponding neurons residing in Johnston’s organ of the fly’s antennae.

**Conclusions:**

These findings provide a novel concept with the neurogenetic basis on the regulation of vertical movement by GMF in the flying animals.

**Electronic supplementary material:**

The online version of this article (doi:10.1186/s13041-016-0235-1) contains supplementary material, which is available to authorized users.

## Background

Geotaxis is a typical innate behavioral response of all living organisms characterized by locomotive activities toward or away from Earth. Particularly, negative geotaxis against Earth’s gravity is prominent in flying animals [1–3]. Remarkable advances have been made in identifying the genes and organs governing the geotactic behaviors using various model organisms, such as the fruit fly, rat, and mouse [3–6]. Especially, two geotaxis genes, *cryptochrome* (*cry*) and *pigment-dispersing factor* (*pdf*) were discovered to be important for the fruit fly’s negative geotaxis [3, 4]. In addition, *pyrexia* (*pyx*), a member of the transient receptor potential (TRP) family, has been also implicated in gravity sensing by Johnston’s organ (JO), the primary organ that senses gravity in *Drosophila* [7, 8].

It is generally accepted that microgravity in a space craft or simulated microgravity on the ground provides suitable experimental conditions to investigate whether gravity provokes geotaxis of living organisms [9–11]. However, this consensus may be flawed because geotactic behaviors under these conditions are confounded by a wide range of artifacts. For instance, geomagnetic field (GMF), one of the bio-effective environmental factors on Earth, is also notably weakened in space [9, 12]. A web of GMF ranging from 25 μT at the magnetic equator to 65 μT at the magnetic poles surrounds Earth [13] and influences animals’ locomotive activities. Indeed, GMF provides migratory animals an important behavioral cue for their horizontal migration [14, 15]. The European robin and eastern red-spotted newt use an inclination compass that provides directional information on the magnetic pole (north) and magnetic equator (south) for north–south migration [15, 16]. Hatchling loggerhead sea turtles exploit longitudinal and latitudinal information, a two-coordinate magnetic map, of GMF to migrate in the North Atlantic Ocean to spawn [17]. Because the trajectories of such flying or swimming animals occur not in planes but in a three-dimensional space, we speculate that GMF may also influence vertical movement.

Interestingly, a recent study showed that electromagnetic field (EMF) interferes with flies’ negative geotaxis [18]. In the study, EMF, of which intensity was heterogeneous and adjusted at an artificially high level of 500 μT, disrupted the gravity-induced negative geotaxis, and this phenomenon was abolished in *cry* mutant flies, suggesting that CRY mediates the effect of EMF [18]. The result strongly implicates that naturally existing magnetic fields including GMF can influence the geotactic behaviors of animals on Earth.

In the present study, we adopted a Helmholtz coil system generating highly refined and reproducible GMF conditions to identify the role of GMF in flies’ geotactic behaviors and the neurogenetic basis for the GMF-modulated geotactic behaviors. Attenuation of GMF to near zero potentiated the gravity-induced negative geotactic behaviors, suggesting the antagonizing effect of GMF on the gravity-induced geotaxis. We also generated particular GMF conditions with increased intensity where flies exhibited robust positive geotaxis. Both the GMF-modulated negative and positive geotactic behaviors required the neural circuit expressing CRY, Pyx and PDF. These results indicate that flies employ the GMF-based neural adjustment to maintain optimal vertical positioning.

## Results

### Potentiated negative geotaxis by near-zero GMF

**Fig. 1:**
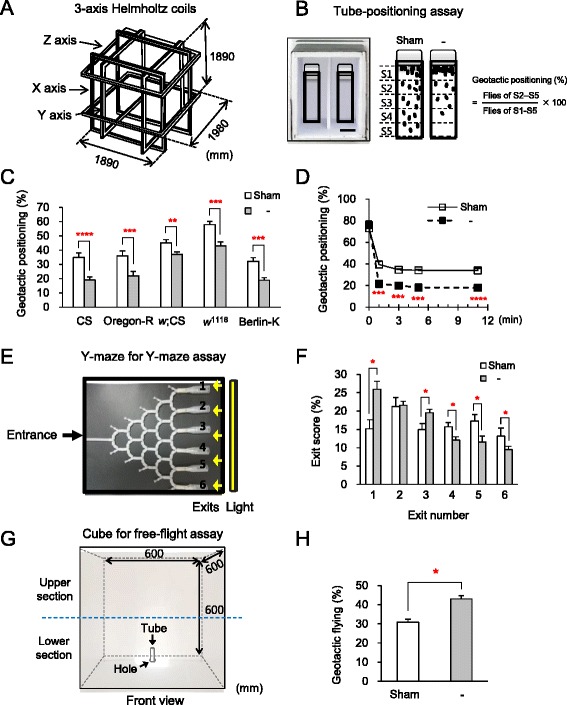
Near-zero GMF potentiates the negative geotactic behaviors in flies. **a** Schematic drawing of the rectangular Helmholtz coil system used to regulate intensity or direction of three GMF vectors by active cancellation. **b** *Left*: Photo of the test cube used for the tube-positioning assay. *Right*: Imaginary drawing of geotactic positioning by the flies under the sham and shield (−) condition in the assay. The geotactic positioning score was calculated at the end point of the test using the following equation: (number of flies at the S2–S5 sections of the test tube equally divided into five imaginary sections/total number of flies) × 100 % (details in Methods). S, section; Sham, ambient GMF; Shield, near-zero GMF. Scale bar: 2 cm. **c** Negative geotactic positioning of fly strains in the shield (−) condition (*n* = 10 trials). Note the significance in all the strains. Error bars: SEM. **, *P* < 0.01; ***, *P* < 0.005; ****, *P* < 0.001 by Student’s *t*-test. **d** Time-course measurements of the positioning score in Canton-S flies under the sham and the shield (−) condition. ***, *P* < 0.005; ****, *P* < 0.001 by Student’s *t*-test. **e** Photo of the six-exit Y-maze used in the assay. For each experiment, 25 ± 2 flies were allowed to enter the maze through the entrance (details in Methods). **f** Exit profiles of the vertical choice Y-maze assay under the sham and shield (−) conditions (*n* = 12 trials). Note the significantly higher scores at the upper exits (1 and 3) under the shield condition compared to the sham condition. Error bars: SEM. *, *P* < 0.05 by Student’s *t*-test. **g** Schematic drawing of the cubic arena used for the free-flight assay (details in Methods). The geotactic flying score was calculated as the percentage of flies that flew to the upper section of the front plane. **h** Geotactic flying scores for the sham and shield (−) in the free-flight assay. Error bars: SEM. *, *P* < 0.05 by Student’s *t*-test (*n* = 15 trials)

To determine if GMF influences the geotactic behaviors of *Drosophila*, we constructed a GMF condition (henceforth, referred to as GMF, unless otherwise indicated) ranging from near-zero to ca. 85 μT which is comparable to the ambient GMF on Earth using a Helmholtz coil system (Fig. 1a). First, we attempted to modulate the intensity of GMF to a near-zero condition (Additional file 1: Table S1), and the tube-positioning assay, a modified version of the tube-climbing assay, was performed in the test cube to measure vertical positioning of flies at Zeitgeber time (ZT) 5 to ZT8 (Fig. 1b and Additional file 2: Figure S1A). A near-zero GMF condition was achieved by cancellation of all three GMF axes, *X*, *Y*, and *Z*, which significantly decreased the geotactic positioning scores (8 %–16 %) in all tested fly strains, Canton-S, white-eyed Canton-S, *w*^*1118*^, Oregon-R, and Berlin-K (*P* < 0.01–0.001), compared to the sham condition (~45 μT in the lab) (Fig. 1c, Additional files 3 and 4) (By the definition described in Methods and Fig. 1b, a decrease in the geotactic positioning score indicates an increase in negative geotaxis and vice versa.). This potentiated negative geotaxis of flies under the near-zero GMF was similarly reproduced at ZT0–ZT2 (Additional file 2: Figure S1B), a circadian period characterized by higher locomotor activities in flies [19]. In addition, the geotactic responses modulated by countervailing fields in Helmholtz coil system was reproduced by passive cancellation of GMF with a permalloy cube made of nickel-iron alloy, a widely used metal for GMF shielding (Additional file 2: Figure S1C, S1D). In a time-course analysis, the geotactic response of Canton-S was fast (approximately 1 min, *P* < 0.005) and was sustained up to 11 min (*P* < 0.001) under the near-zero GMF (Fig. 1d and Additional file 4). Based on these robust and consistent responses, the Canton-S line was primarily used in subsequent experiments.

In another widely used geotaxis assay employing a vertical choice Y-maze [4] (Fig. 1e), more flies left the maze through the upper exits (1 and 3) than the lower exits (4–6) under the near-zero GMF than the ambient GMF (*P* < 0.05) (Fig. 1f), confirming that attenuation of GMF strengthens the negative geotactic nature of flies. As a control, the exit profiles of flies through the vertical maze versus horizontal maze in the ambient GMF were reproduced as previously described [4] (Additional file 2: Figure S1E). To further confirm the near-zero GMF-induced negative geotaxis in a more natural setup, a free-flight assay in which flies could freely fly in a cubic arena was also conducted (Fig. 1g). Consistent with the above results, the near-zero GMF elicited flying at higher elevations (*P* < 0.05) (Fig. 1h). Collectively, these results strongly suggest that GMF antagonizes negative geotaxis in *Drosophila*.

### Positive geotaxis induced by modulated GMF

**Fig. 2:**
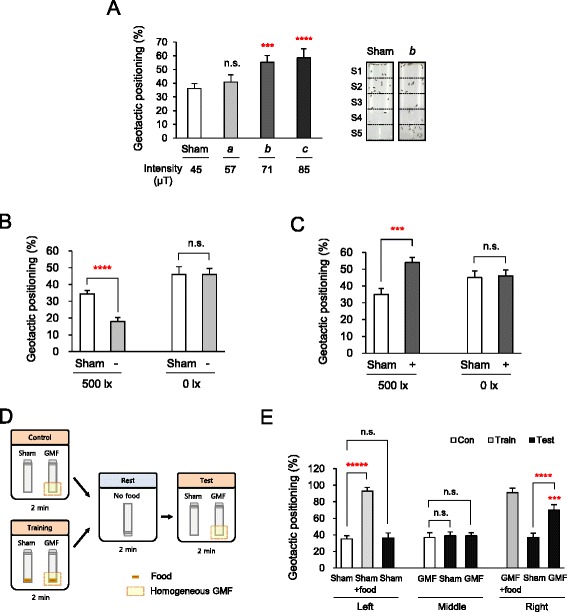
Modulated GMF with increased intensity induces positive geotaxis. **a** *Left*: Comparisons of the geotactic positioning under the GMF conditions with modulated intensities. A positive geotaxis was induced in conditions *b* and *c*. Error bars: SEM. n.s.: not significant. ***, *P* < 0.005; ****, *P* < 0.001 by ANOVA Tukey’s test. (*n* = 10 trials). *Right*: A representative image of geotactic positioning under the sham and *b*, respectively. **b**, **c** Comparisons of the geotactic positioning of the wild-type flies between light (500 lx) versus dark (0 lx) conditions under the near-zero GMF condition and the GMF condition *b* in Fig. 2a, respectively. Note the increase of positioning score of the sham samples under the dark condition. −; near-zero GMF, +; GMF condition *b*. Error bars: SEM. n.s.: not significant. ***, *P* < 0.005; ****, *P* < 0.001 by Student’s *t*-test. **d** Schematic drawing of the associative learning assay using food as an unconditioned stimulus and the non-geotactic GMF as a conditioned stimulus. The GMF denotes the non-geotactic GMF *a*. Note that the test tubes were inverted during the rest and the GMF was provided from the bottom side of the tubes. The homogeneous space for the GMF is marked as dashed rectangles. **e** Comparisons of the geotactic positioning induced by the control, trained, and test conditions in the presence of the ambient GMF condition (Sham) or the GMF stimulus, or either one associated with food. GMF, the non-geotactic GMF condition *a* in Fig. 2a. Food, the rearing diet. Error bars: SEM. n.s.: not significant. ****, *P* < 0.001; *****, *P* < 0.0001; ***, *P* < 0.005 compared to the sample of the middle group, associated with the GMF alone and then tested under the same GMF, by ANOVA or Student’s *t*-test (*n* = 10 trials)

A growing body of evidence suggests that the individual components of GMF provide fundamentally different information to magnetosensitive animals. The vector of GMF provides directional information, whereas the intensity and/or inclination provide positional information for horizontal movement [15]. However, GMF has not been previously suggested as a cue for vertical movement of animals such as geotactic behaviors. Since the near-zero GMF potentiated negative geotaxis as shown in Fig. 1, we next asked whether any GMF conditions with increased intensity make flies actively move downward to the bottom *per se*. Therefore, we examined the geotactic behaviors of flies in response to specific GMF conditions with increased intensity up to 85 μT (Additional file 5: Table S2). Interestingly, we observed robust positive geotactic responses of the flies under the GMF conditions at 71 μT (*b* in Fig. 2a) and 85 μT (*c* in Fig. 2a) (*P* < 0.005 and < 0.001, respectively). To rule out possible influence from the experimental setup (see Fig. 1b, *left*), we carried out a control experiment by rotating the test cube 90° counterclockwise in the horizontal plane. The geotactic scores were comparable between the two directions of the test cube under the near-zero GMF and the positive geotactic GMF condition *b* (Additional file 6: Figure S2A). These results demonstrate that flies can move downward, i.e., positive geotactic, under the GMF conditions with increased intensity.

It has been known that magnetosensitive movements in *Drosophila* are mediated by a blue light-dependent manner [20, 21]. We evaluated the possibility that wild-type flies exhibit differential geotactic behaviors in response to light and dark conditions. Interestingly, flies did not exhibit substantial changes in geotactic behaviors under the near-zero GMF and the positive geotactic GMF condition *b* in Fig. 2a in the dark condition (0 lx) (Fig. 2b and c), indicating that light is required for GMF-modulated geotaxis. In addition, the potentiated negative geotaxis at short wavelengths (400–420 nm) (*P* < 0.005) was comparable to that at full spectrum (*P* < 0.005) (Additional file 6: Figure S2B), demonstrating that blue light is sufficient for the GMF-modulated geotaxis.

Next, we examined whether the GMF-modulated positive geotaxis is biologically relevant for a stereotyped fly behavior. To address this question, we conducted an associative learning experiment in which the fly memory on the food located at the bottom of a test tube was associated with the memory on a non-geotactic GMF condition (Fig. 2d). The non-geotactic GMF condition *a* was demonstrated to function as a neutral stimulus for geotactic responses (Fig. 2a). If starved flies successfully learned to associate the appetite-driven stimulus with the non-geotactic GMF, the flies would exhibit a positive geotactic response upon the sole presentation of the non-geotactic GMF without food. To associate the appetite-driven stimulus with the non-geotactic GMF condition, a group of starved flies was initially trained for 2 min under non-geotactic GMF conditions with food. After a 2 min-rest with the test tube upside-down to eliminate any potential bias for the bottom of the tube, the starved flies were subjected to the non-geotactic GMF condition without food for the test. As a control, parallel experiments were performed in which no food was supplied during the initial training (Fig. 2d). Strikingly, the flies trained by the non-geotactic GMF stimulus with food (Fig. 2e, *right*) exhibited significant positive geotactic responses under the non-geotactic GMF alone (*P* < 0.005) (Fig. 2e, *right*), but no remarkable behavioral changes under the sham condition (Fig. 2e, *right*). By contrast, the control flies for which no food was associated with the non-geotactic GMF stimulus (Fig. 2e, *middle*) did not exhibit a noticeable positive geotactic response under the non-geotactic GMF condition (Fig. 2e, *middle*). Collectively, these results demonstrate that GMF can function as a biologically relevant behavioral cue for vertical movement in flies, particularly when it is associated with the appetitive stimulus that is compulsory for survival in the wild.

### Necessity of CRY, PDF and Pyx pathways in positive geotaxis

**Fig. 3:**
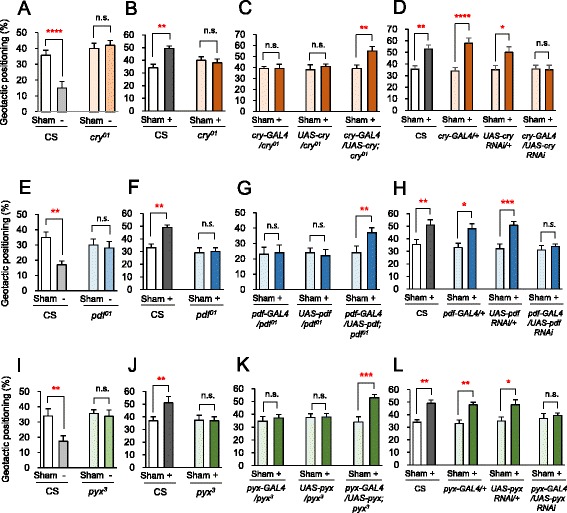
CRY, PDF, and Pyx pathways are required for GMF-modulated geotaxis. **a**, **e**, **i** Comparisons of the geotactic positioning of wild-type and the CRY-, PDF- and Pyx-deficient flies, respectively, under the negative geotactic GMF condition. Error bars: SEM. n.s.: not significant. **, *P* < 0.01; ****, *P* < 0.001 by Student’s *t*-test. **b**, **f**, **j** Comparisons of the geotactic positioning of the wild-type and null mutant flies for *cry*, *pdf*, and *pyx*, respectively, under the positive geotactic GMF condition. Error bars: SEM. n.s.: not significant. **, *P* < 0.01 by Student’s *t*-test. **c**, **g**, **k** Comparisons of the geotactic positioning of the flies mutant for *cry*, *pdf*, and *pyx*, and the flies in which these genes were genetically restored in the mutant background, respectively. Error bars: SEM. n.s.: not significant. **, *P* < 0.01; ***, *P* < 0.005 by Student’s *t*-test. **d**, **h**, **l** Comparisons of the geotactic positioning of the wild-type flies, control flies (*GAL4* transgene alone, *UAS-RNAi* alone), and flies with knockdown of *cry*, *pdf*, and *pyx* transcripts using the gene-specific *GAL4* driver, respectively. Error bars: SEM. n.s.: not significant. *, *P* < 0.05; **, *P* < 0.01; ***, *P* < 0.005; ****, *P* < 0.001 by Student’s *t*-test. For all the data, *n* = 10 trials

To characterize the biological mechanisms underlying the GMF-modulated positive geotaxis, we evaluated the requirements of the signaling pathways that convey the environmental magnetoreceptive information toward the locomotive organs. *cry* was identified as a geotaxis gene through behavioral genetic screening, and an inverse correlation between the *cry* transcript level and negative geotaxis was observed [3]. In addition, given the observed results in Fig. 2b, c and Additional file 6: Figure S2B and that CRY is a light-responsive protein [20, 21], we hypothesized that CRY is involved in the GMF-induced geotaxis. To test this hypothesis, CRY-deficient *cry*^*01*^ mutant flies [20] backcrossed into the wild-type Canton-S background were used to enable the comparison of their geotactic behaviors with those of Canton-S flies as controls. First, we observed that CRY-deficient flies displayed abrogated negative geotactic behaviors that were normally potentiated by the near-zero GMF (Fig. 3a). To check the positive geotactic responses of the mutants, we used the same GMF condition *b* (Intensity = 71 μT) that elicited positive geotactic responses in Fig. 2a. In this condition, CRY-deficient flies exhibited impaired positive geotactic responses compared to wild-type controls, and CRY expression-rescued flies exhibited normal positive geotactic responses (Fig. 3b and c, respectively). In addition, RNAi knockdown of *cry* using the *cry-GAL4* driver completely abolished the positive geotactic responses compared to wild-type and other control flies (Fig. 3d). Moreover, CRY-deficient flies were defective in the near-zero GMF-induced negative geotaxis and the CRY expression-rescued flies recovered the geotactic behavioral phenotype (Additional file 7: Figure S3A). Similarly, the impaired geotactic response in *cry*-knockdown flies was restored by the recovering of the expression of CRY in the mutant flies (Additional file 7: Figure S3B).

Previous studies suggested that *pdf* and *pdf receptor* (*pdfr*) play crucial roles in geotaxis [3, 22]. Thus, we examined whether PDF also plays a role in the GMF-modulated geotactic behaviors using *pdf*^*01*^ mutant flies, a PDF-deficient line [23]. Interestingly, *pdf*^*01*^ mutant flies were defective in both the near-zero GMF-induced negative geotactic and positive geotactic responses compared to wild-type controls (Fig. 3e and f). Genetic restoration of PDF expression in the mutants fully rescued the defect in both geotactic responses (Fig. 3g and Additional file 7: Figure S3C). Supporting these results, specific knockdown of *pdf* using *pdf-GAL4* driver also impaired both the near-zero GMF-induced negative geotaxis and positive geotactic responses (Fig. 3h and Additional file 7: Figure S3D). Together, these results demonstrate that PDF is required for the GMF-modulated geotactic behavior in flies.

We next examined the GMF-modulated geotactic responses in a *pyx*-deficient mutant line because Pyx encoded by *pyx* belongs to the TRP channel family and critically contributes to gravity sensing in the *Drosophila* JO [7, 8]. The mutant flies exhibited impaired GMF-modulated positive geotaxis and near-zero GMF-induced negative geotaxis (Fig. 3i and j, respectively), and both of the impaired geotactic responses were successfully recovered by genetic rescue of *pyx* (Fig. 3k and Additional file 7: Figure S3E, respectively). Similarly, RNAi knockdown of *pyx* abrogated the positive geotactic positioning and near-zero GMF-induced negative geotaxis (Fig. 3l and Additional file 7: Figure S3F, respectively). These results consistently suggest that Pyx is required for the GMF-modulated geotactic behaviors.

**Fig. 4:**
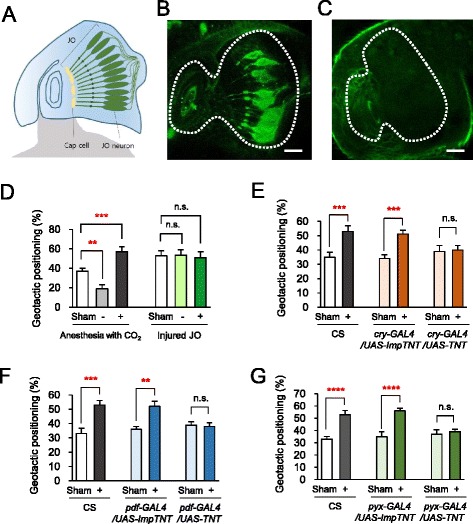
*cry-*, *pdf-* and *pyx-GAL4* expressing neurons are necessary for the GMF-induced geotactic positioning. **a** Schematic representation of JO in the second antennal segment. **b**, **c** Expression of GFP in *cry-GAL4*/*UAS-mCD8::GFP* and *pdf-GAL4*/*UAS-mCD8::GFP*, respectively. Scale bar: 10 μm. **d** The geotactic positioning of JO-injured flies. The second antennal segments of flies were pinched with fine forceps under CO_2_ anesthesia 24 h before the tube-positioning assay. Anesthetized flies without JO injury were controls. Error bars: SEM. n.s.: not significant. **, *P* < 0.01; ***, *P* < 0.005 by ANOVA (*n* = 10 trials). **e**, **f**, **g** The geotactic positioning of the flies with targeted inhibition of the neurons expressing CRY, PDF, and Pyx, respectively, by expressing TNT under the positive geotactic GMF condition. Control flies expressed impTNT. Error bars: SEM. n.s.: not significant. **, *P* < 0.01; ***, *P* < 0.005; ****, *P* < 0.001 by Student’s *t*-test. For all the data, *n* = 10 trials

Having shown that CRY, PDF and Pyx genes are required for the GMF-modulated geotactic behaviors, we investigated the neuroanatomical loci critical for the behaviors. We paid first attention to JO that is comprised of specialized neurons and cap cells for perceiving mechanical stimuli and sensing gravity for normal geotaxis (Fig. 4a) [7, 8]. Intriguingly, Pyx is expressed in the cap cells in JO [8] and a batch of prominent CRY neurons morphologically corresponding to JO neurons was visualized (Fig. 4b). However, we found no evidence for PDF neurons located in JO (Fig. 4c). Prior to testing the necessity of these neurons in the GMF-modulated geotaxis, we confirmed whether JO is indeed critical for the GMF-modulated geotactic responses. To achieve this, we physically injured JO in the second segment of antenna [8] and checked the geotactic responses. As a result, we observed that the flies with injured JO exhibited severely impaired GMF-modulated geotactic behaviors (Fig. 4d). As a comparison, we also cut off flies’ wings [24] and examined the injured flies’ geotactic behaviors, but found no significant difference between the injured flies and controls (Additional file 8: Figure S4A), substantiating the hypothesis that JO is critical locus for the GMF-modulated geotaxis in flies. To next examine if the CRY, PDF and Pyx neurons in JO are required for the GMF-modulated geotactic responses, we targeted expression of tetanus toxin (TNT) to silence each of these neurons expressing CRY, PDF and Pyx using their specific *GAL4* drivers. Interestingly, silencing CRY, PDF and Pyx neurons all abrogated the GMF-modulated positive geotaxis (Fig. 4e-g). Also, silencing these neurons abolished the near-zero GMF-induced negative geotaxis (Additional file 8: Figure S4B-S4D). Taken together, these results demonstrate that a neural circuit expressing CRY, PDF, and Pyx is required for the GMF-modulated geotactic behaviors and suggest that the circuit-mediated sensory processes for the GMF-modulated geotactic responses may occur in JO.

### Johnston’s organ-specific role of CRY and Pyx pathways

**Fig. 5:**
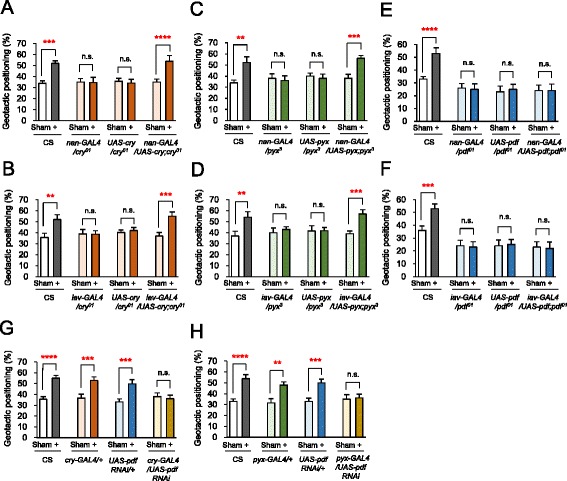
CRY and Pyx function in Johnston’s organ for GMF-modulated positive geotaxis. **a**, **c**, **e** Comparisons of the geotactic positioning of wild-type flies, the fly mutants for *cry*, *pyx* and *pdf*, and the mutant flies in which these genes were genetically restored using *nan-GAL4* driver, a JO-specific *GAL4* driver, respectively. Error bars: SEM. n.s.: not significant. **, *P* < 0.01; ***, *P* < 0.005; ****, *P* < 0.001 by Student’s *t*-test. **b**, **d**, **f** Comparisons of the geotactic positioning of wild-type flies, the fly mutants for *cry*, *pyx* and *pdf*, and the mutant flies in which these genes were genetically restored using *iav-GAL4* driver, another JO-specific *GAL4* driver, respectively. Error bars: SEM. n.s.: not significant. **, *P* < 0.01; ***, *P* < 0.005 by Student’s *t*-test. **g**, **h** Comparisons of the geotactic positioning of wild-type flies, control flies (*GAL4* driver alone, *UAS-pdf RNAi* alone), and the flies with RNAi knockdown of *pdf* using *cry*-*GAL4* or *pyx-GAL4* driver, respectively. Error bars: SEM. n.s.: not significant. **, *P* < 0.01; ***, *P* < 0.005; ****, *P* < 0.001 by Student’s *t*-test. For all the data, *n* = 10 trials

To determine whether the functions of CRY, PDF and Pyx in JO are critical for the GMF-modulated geotactic responses, we selectively restored the gene expression of *cry*, *pdf* and *pyx* in the fly mutants of these genes using two JO-specific *GAL4* drivers, *nanchung* (*nan*) and *inactive* (*iav*) [8]. Supporting the hypothesis, JO-specific expression of exogenous *cry* using these two different *GAL4* drivers in CRY-deficient flies completely rescued the impaired GMF-modulated geotactic behaviors (Fig. 5a, b, Additional file 9: Figure S5A and S5B). Similarly, the defective GMF-modulated geotactic responses in Pyx-deficient flies were also rescued by JO-specific expression of exogenous *pyx* (Fig. 5c, d, Additional file 9: Figure S5C and S5D). These results indicate that CRY and Pyx function in JO to mediate the GMF-modulated geotactic behaviors.

Strikingly, however, JO-specific expression of PDF was not sufficient to rescue the defective GMF-modulated geotactic responses of *pdf* mutant flies (Fig. 5e, f, Additional file 9: Figure S5E and S5F). This result is in consistence with the notion that PDF is not expressed in JO (Fig. 4c), suggesting that the PDF neurons in the remaining body parts are responsible for the GMF-modulated geotactic responses. Intriguingly, we found that RNAi knockdown of *pdf* in CRY- or Pyx-expressing cells impaired the GMF-modulated geotactic responses (Fig. 5g, h, Additional file 9: Figure S5G and S5H). Taken together, these data suggest that PDF functions in the GMF-modulated geotactic responses elsewhere in the CRY- and Pyx-expressing cells.

## Discussion and conclusions

As revealed by our work, GMF serves as a driving force for geotactic behaviors; attenuating it to near zero potentiated the gravity-induced negative geotaxis, whereas specific GMF conditions with increased intensity elicited robust positive geotaxis. Consistently, we observed that flies locate themselves at higher positions upon exposure to a negative geotactic GMF condition in a free-flight experimental setup. Based on these results, we hypothesize that the bipolar geotactic responses modulated by GMF would in turn affect a flying animal’s vertical movements.

Our data demonstrated that the GMF-modulated geotaxis is a genuine behavior-oriented response that utilizes specific tissues (i.e. JO), neural circuits and genes to integrate the surrounding GMF information. To investigate whether flies sense the existence of GMF and learn to associate it as a behavioral cue, we trained flies using food located at the bottom of a tube to make them move downward in the presence of a non-geotactic GMF to determine if flies would move downward due to the memory of the GMF previously associated with food. Surprisingly, flies indeed moved downward in the presence of a non-geotactic GMF even in the absence of food, clearly demonstrating that GMF could act as a geotactic behavioral cue for *Drosophila*.

JO-selective genetic restoration of *cry* and *pyx* successfully rescued the impaired GMF-modulated geotactic responses of the fly mutants of these genes, whereas JO-specific *pdf* expression was insufficient to rescue the defective geotactic responses of PDF-deficient mutants (Fig. 5 and Additional file 9: Figure S5). These results raise two intriguing questions. 1) Do other tissues mediate the GMF-modulated geotactic responses? 2) If so, what is the hierarchy among JO and these tissues? A recent study attempted to selectively restore CRY expression in different tissues of *cry* mutant flies and tested their geotactic behaviors in response to electromagnetic field (EMF) [18]. The study found that selective restoration of CRY in three different tissues, including JO, dorsal lateral neurons (LN_d_s), and particular regions in the eye, rescued the disturbed climbing responses induced by EMF to varying degrees. Consistent with the involvement of multiple tissues in the EMF-modulated geotactic responses, we also conclude that both *nan*/*iav*-positive cells in JO and PDF-positive CRY-expressing cells in different tissues are important for the GMF-modulated geotactic responses. Indeed, CRY and PDF are co-expressed in a subset of clock neurons in the brain, such as small ventral lateral neurons (s-LN_v_s) and large ventral lateral neurons (l-LN_v_s), and thus form the interfacial neural circuits for circadian output pathways [25, 26]. In addition, circadian rhythm signals from CRY-expressing clock neurons are projected onto the PDF-PDFR signaling circuits and thus are eventually propagated toward locomotor organs such as the legs and/or wings [26–28]. In fact, the circadian locomotor activity of Canton-S flies was affected by EMF, and this effect required CRY [29]. Therefore, it is conceivable that the environmental GMF sensed by the CRY-mediated pathway in JO converges on the subset of clock neurons in the brain that controls locomotive activities to execute the complete process of the GMF-modulated geotactic responses.

What is the biological significance of the GMF-modulated geotaxis in nature? GMF is critical for the high accuracy of long-distance travel by migratory animals [14, 15, 17]. We speculate that the importance of GMF as a behavioral cue may be attributable to its consistency, in contrast to olfaction and vision which are locally formed and weakened or distorted by weather changes. For example, navigating animals may imprint sets of the GMF parameters for feeding sites along the past migratory routes via food-GMF associative learning, which allows them to secure fueling sites for subsequent journeys. Supporting this notion, a study presented evidence that the migratory songbird thrush nightingale (*Luscinia luscinia*) exploits GMF for locating food sources before crossing the Sahara Desert, a large ecological barrier [30]. In addition, a theory on radical pair mechanism (RPM) provided the genetic basis for sensing GMF by CRY. According to the RPM theory, the three-dimensional pattern of magnetoreception mediated by CRY provides a spherical coordinate system in the brain, and that can elicit directional and spatial responses of animals in three dimensions [31]. These studies support our finding that GMF would serve as an important behavioral cue for animal’s vertical movements during the navigation in nature. Further investigation of how animals sense and utilize GMF components, e.g., total intensity, inclination, declination, etc. will lead us to a better understanding of the behaviors of magnetotactic animals on Earth.

## Methods

### Fly stocks and genetics

All flies were reared with standard cornmeal-yeast-agar diet at 25 °C, 60 % relative humidity, in a 12 h light/12 h dark cycle under a full-spectrum fluorescent light that was turned on at 09:00 (local time), and the ambient geomagnetic field (GMF; total intensity = 44.9 μT, *X* (North–south) = 32.2 μT, *Y* (East–west) = −5.8 μT, *Z* (vertical to ground) = 30.8 μT). Fly strains of Canton-S, Oregon-R, *w*^1118^, Berlin-K, *cry-GAL4*^*16*^, *nan-GAL4*, *iav-GAL4*, *UAS-mCD8::GFP*, *UAS-cry RNAi* (BL25859), and *UAS-pyx RNAi* (BL31297) were provided by the Bloomington *Drosophila* Stock Center (Indiana University, Bloomington, IN, USA). *UAS-pdf RNAi* (v4380) was provided by Vienna *Drosophila* RNAi Center (Vienna, Austria). Other flies were gifts: *cry*^*01*^ flies [20] from Dr. S. M. Reppert (Univ. of Massachusetts, USA); *pdf*^*01*^ [23] from Dr. J. H. Park (Univ. of Tennessee, USA); *UAS-TNT* and *UAS-ImpTNT* [32] from Dr. C. J. O’Kane (Univ. of Cambridge, UK), and *pdf-GAL4* [19] from Dr. J. Choe (KAIST, Korea); *pyx-GAL4* [8] from Dr. F. N. Hamada (Cincinnati Children’s Hospital Medical Center, USA); *UAS-cry* [33] from Dr. E. Rosato (Univ. of Leicester, UK); *UAS-pdf* [19] from Dr. P. H. Taghert (Washington Univ., USA). *cry*^*01*^, *pdf*^*01*^, and *pyx*^*3*^ were outcrossed to Canton-S background for 6–8 generations. For restoring the mutant phenotype using GAL4-UAS system in each *cry*^*01*^, *pdf*^*01*^, or *pyx*^*3*^ mutant background, *pyx-GAL4* and *iav-GAL4* were recombined onto the third chromosome carrying each mutant allele. There was no abnormal phenotype in appearances and life cycle during the development of fly strains throughout the experiments, except low oviposition rate in PDF-deficient files.

### Regulation of GMF

A rectangular Helmholtz coil system (Fig. 1a) consisting of three pairs of parallel coils arranged orthogonally for three axes was constructed based on our previous Helmholtz coil system [34–36], and it was used to modulate the intensity of three GMF vectors by active cancellation. The coil for X axis (North–south) was aligned with true geographic north so that Y-coil has a room for contributing to modulate *Y* vector of GMF. The position of geographic north was determined using the local declination value (−7.53 at the time of set-up) of Daegu city outside the building where the experiments were performed. Using this set-up, we intended to maximize the diversity of GMF conditions close to the real world for testing flies’ geotactic behaviors. Average dimensions of the coils were 1,890 × 1,890 mm, 1,890 × 1,800 mm, and 1,980 × 1,980 mm, for the X, Y, and Z axes, respectively. Coils for each axis comprised two bundles (1 mm in diameter enameled Cu wire, 175-turns/bundle) on an open non-magnetic aluminum frame aligned in parallel with each other, with effective distances of 1,029 mm, 980 mm, and 1,078 mm for the X, Y, and Z axes, respectively. A pair of coils for each axis was connected to an adjustable DC power supply (E3631A; Agilent Technologies, Santa Clara, CA, USA). GMF was measured using a 3-axis gaussmeter (MGM 3AXIS; ALPHALAB, West Salt Lake City, UT, USA), and homogeneity of the GMF in the sample space was measured as 99 %, 95 %, and 90 % for the tube-positioning assay, Y-maze assay, and free-flight assay, respectively. The modified GMF parameter conditions and the intensity of fluorescent light (500 lx unless otherwise mentioned) were indicated in figure legends or table captions accordingly. The ambient power frequency of the 60-Hz magnetic field was less than 3 μT, as measured by a gaussmeter (TES 1390; TES Electrical Electronic, Taipei, Taiwan). Throughout the experiments, 60 Hz electric field across the assay area was measured using (3D NF Analyzer NFA 1000; Gigahertz Solutions, Fürth, Bayern, Germany). The difference of electric field between experimental conditions was negligible; sham (1.22 V/m), cancellation of *X*, *Y*, and *Z* (1.21 V/m), condition *a* (1.21 V/m), *b* (1.22 V/m), and *c* (1.22 V/m).

To decrease GMF to near zero (indicated as – in figures), we cancelled the intensity of each axis of the ambient GMF by countervailing the each axis using the coil system (Figs. 1c, d, f, h, 2b, 3a, e, i, 4d, Additional file 2: Figure S1B, Additional file 6: Figure S2A-B, Additional file 7: Figure S3A-F, Additional file 8: Figure S4A-D and Additional file 9: Figure S5A-H). Conversely, positive geotaxis was induced by strengthening the total intensity as appeared in b and c of Fig. 2a. The strengthening condition was indicated as + in Figs. 2c, 3b-d, f-h, j-l, 4d-g, 5a-h and Additional file 8: Figure S4A.

Exceptionally, passive shielding was used for Additional file 2: Figure S1D. This shielding condition was achieved by conducting behavioral experiments in a double-layered permalloy (0.5-mm thick) cube (180 × 100 × 140 mm, length × width × height) (Additional file 2: Figure S1C) with one open-side for handling of samples and recording of flight behaviors. The passive shielding experiment also showed similarly potentiated negative geotaxis comparable to the countervailing method using the coil system (Additional file 2: Figure S1D). The cancellation efficiency for total intensity was ca. 36-fold (69-, 71-, and 33-fold for the X, Y, and Z axes, respectively), and the homogeneity was about 95 % in the sample space.

### Geotaxis assay

All the experiments using the coil were conducted in a temperature-controlled room kept at 25 °C and the temperature across the assaying area was monitored using thermometers (USB Datalogger 98581; MIC Meter Industrial Company, Taichung City, Taiwan). The fly samples were set inside the coils 3 s after turning on the current. The tube-positioning assay was performed mostly between ZT5 and ZT8 as well as ZT0 and ZT2 in a separate experiment according to a previous study [37] with some modifications. Prior to the assay, flies (1 ~ 3-day-old) taken from the rearing incubator were put into a pooled flask (180 mL) and accommodated for 10 min. The flies (45 ± 2) were then transferred into a transparent polypropylene test tube (20 × 850 mm, diameter × height) sealed by a cotton plug at the orifice without anesthesia. The test tubes with the flies were kept inverted on the top for 1 min and then gently placed with the bottom down inside either a cube (180 × 100 × 140 mm, length × width × height) (Fig. 1b and Additional file 2: Figure S1A) located at the center of the Helmholtz coil. Vertical positioning of the flies in the test tubes was video-recorded for 11 min and quantified as the “geotactic positioning score” using the following equation: (number of flies at the lowest four sections of the test tube that was equally divided into five imaginary sections/total number of flies) × 100 % (see Fig. 1b). Other alternative equations that included ‘the lowest three sections’ and ‘the lowest two sections’ instead of ‘the lowest four sections’ showed similar scores (Pearson correlation [*R*] values were 0.98 and 0.96, respectively). The calculation was performed on the five consecutive captured photos at 5 s interval, and the average of the scores from the five photos was used as a data point.

For the removal of the JO or wings in Fig. 4d and Additional file 8: Figure S4A, the JO was injured by pinching the second antennal segments with fine forceps [8] or wings were cut off at the hinge region with micro-scissors [24] under anesthesia with CO_2_ [8, 24]. The injured flies were accommodated to the rearing incubator for 24 h before the tube-positioning assay (10 flies/test), and then the assay was performed.

The Y-maze assay was adopted as an alternative geotaxis assay with some modifications [3, 4]. The six-exit maze was constructed from clear polypropylene T- and Y-shaped connectors 4 mm in diameter (Kartell, Noviglio, Italy), and a 4-mm-bore non-toxic silicon tube (Cole-Parmer, Vernon Hills, IL, USA) (Fig. 1e). For collection of flies at the exits, a semi-transparent 1-ml plastic tip used for micropipettes was connected to each exit. Each tip was jointed with a tapered 15-ml clear polypropylene conical tube to prevent flies from reentering the maze through the exit. The prepared maze was fixed on a transparent acryl plate for rigidity and placed at the center of the Helmholtz coils. The Y-maze assay was performed between ZT0 and ZT2. For each experiment, 25 ± 2 flies were transferred from a pooled flask to a 15-ml clear polypropylene conical tube (feeding tube) and allowed to enter the maze through a 10-cm-long silicon tube linking the feeding tube and the entrance of the maze. The flies were kept there for 30 min until at least 90 % of them were collected. Light was placed parallel to all exits, and the intensity of light was 350 lx and 500 lx at the entrance and all exits, respectively. The exit score for each exit was calculated as (number of flies collected at each exit/total number of flies collected at all exits) × 100 %. Exit profiles were compared between the sham (the ambient GMF) and near-zero GMF conditions (total intensity, 0.015 ± 0.006 μT), in which the plane of the maze was vertical to the ground. As a control, the exit profile of the sham-horizontal maze was compared to that of the sham-vertical maze.

The free-flight assay was performed to determine the effect of GMF-induced modulation on free flying behavior of flies in a cubic arena (0.6 × 0.6 × 0.6 m) (Fig. 1g). The five planes of the arena were made of pale white board, whereas the front plane was a transparent film through which light could shine and flying behavior could be recorded. A fluorescent light lamp was placed 5 cm above the bottom line of the arena, and the light intensity was 500 lx at the point of departure. The assay was performed between ZT0 and ZT2. For each experiment, 30 ± 2 flies were transferred from the pooled flask to a 15-ml clear polypropylene conical tube. Upon removing the cap, the tube was instantly intruded 6 cm into the arena through a hole (20 mm in diameter) at the rear-center region of the bottom, and the flies were allowed to crawl up to the orifice and fly freely. Flying behavior was recorded for 2 min. The GMF inside of the arena was modulated as indicated in Fig. 1h. Flying score was calculated as (number of flies flown onto the upper section of the front plane/total number of flies flown onto the whole section of the front plane) × 100 %. As a control, the flying profile under the sham conditions (the ambient GMF) was determined.

The associative learning assay was carried out according to the scheme depicted in Fig. 2d. One to two-day-old Canton-S flies were transferred to an empty flask containing Whatman paper soaked with distilled water and starved for 24 h; they were then placed in another empty flask containing no water for 6 h before training. In each experiment, 20 ± 2 flies were used. For the training group, flies were loaded into a training tube containing food, the same rearing diet, either in the sham or the non-geotactic GMF for 2 min. The trained flies were transferred to a test tube that was inverted during the 2-min rest and subsequently tested for 2 min. In the control experiments, flies were processed by the same procedure without food in a tube. For the test, the tube-positioning assay was conducted to measure geotactic behavior of the trained or control flies under the sham or the non-geotactic GMF condition. The trained and control flies were experimented consecutively. The non-geotactic GMF was generated using a home-made one-axis square Helmholtz coil (250 × 250 × 100 mm, 1,000 turns, 1 mm in diameter enameled Cu wire) on open acrylic frame. The coils were positioned perpendicular to a test tube at its base; homogeneity of the non-geotactic GMF intensity at the lower one-third of test tube was 95 % (see Fig. 2d).

### Statistical analysis

Statistical analyses were performed by Student’s *t*-test or one-way analysis of variance (ANOVA) Tukey’s test using Origin software (San Clemente, CA, USA). Statistical values are presented as mean ± standard error of the mean (SEM). In all analyses, *P* < 0.05 is regarded as significant. All experiments were repeated at least 10 times.

### Ethics approval and consent to participate

Not applicable

### Consent for publication

Not applicable

### Availability of data and material

Supporting data are found in the supporting materials; 5 supporting figures, 2 supporting tables, 2 supporting videos, and one striking image.

## Additional files

Additional file 1: Table S1. GMF parameters of the near-zero GMF condition in Fig. 1. Values of *X*, *Y*, and *Z* intensity are the means of 10 trials for each condition. Total intensity, was calculated using the formula $\sqrt{X^2+{Y}^2+{Z}^2}$ [13]. Sham: ambient GMF, −: near-zero GMF condition. (DOC 27 kb) Additional file 2: Figure S1. Near-zero GMF-induced geotactic response under different ZTs and the comparison of vertical choice vs. horizontal choice Y-maze assays. (A) Photos of the test cube (180 × 100 × 140 mm, length × width × height) used for the tube-positioning assay. (B) Comparisons of the geotactic positioning of wild-type flies under the sham and cancellation conditions during ZT0 to ZT2 (*n* = 10 trials). Error bars: SEM. *, *P* < 0.05 by Student’s *t*-test. (C) Photos of the double-layered permalloy (0.5 mm thick) cube (180 × 100 × 140 mm, length × width × height) used for the attenuation of GMF by passive cancellation. (D) Comparisons of the geotactic positioning of wild-type flies under the sham and passive cancellation conditions during ZT5 to ZT8 (*n* = 10 trials). Error bars: SEM. *, *P* < 0.05 by Student’s *t*-test. (E) Comparisons of the exit profiles of wild-type flies making vertical choice versus horizontal choice in the Y-maze assays under the sham condition (*n* = 12 trials). Error bars: SEM. *, *P* < 0.05; **, *P* < 0.01 by Student’s *t*-test. (PDF 81 kb) Additional file 3: Video. A representative normal behavior of Canton-S flies in the tube-positioning assay under the sham condition (the ambient GMF) (Fig. 1c). (AVI 6775 kb) Additional file 4: Video. A representative negative geotactic behavior of Canton-S flies in the tube-positioning assay under the cancellation condition (the near-zero GMF) (Fig. 1c). (AVI 6674 kb) Additional file 5: Table S2. GMF parameters of the positive geotactic GMF conditions in Fig. 2a. Values of *X*, *Y*, and *Z* intensity are the means of 10 trials for each condition. Total intensity, was calculated using the formula $\sqrt{X^2+{Y}^2+{Z}^2}$ [13]. *a*, *b*, and *c* are the GMF conditions under which the geotactic positioning was measured, respectively. Note that *b* and *c* were positive geotactic GMF conditions. (DOC 28 kb) Additional file 6: Figure S2. Comparisons of the geotactic positioning under different direction of the test cube and various light wavelengths. (A) Comparison of geotactic positioning of flies between two different directions, i.e., control and counterclockwise (90°) for the test cube under sham, the shield (−), and the positive geotactic GMF (+). −; near-zero GMF, +; GMF condition *b*. Error bars: SEM. **, *P* < 0.01; ***, *P* < 0.005 by ANOVA Tukey’s test (*n* = 10 trials). (B) The light intensity was 500 lx in all the experimental conditions. Error bars: SEM. n.s.: not significant. ***, *P* < 0.005 by Student’s *t*-test. (*n* = 10 trials). (PDF 23 kb) Additional file 7: Figure S3. CRY, PDF and Pyx pathways are required for the near-zero GMF-induced negative geotaxis. (A, C, E) Comparisons of the geotactic positioning of the CRY-, PDF- and Pyx-deficient flies rescued with their coding genes (*cry-GAL4*/*UAS*-*cry;cry* ^*01*^, *pdf-GAL4*/*UAS*-*pdf;pdf* ^*01*^ and *pyx-GAL4*/*UAS*-*pyx;pyx* ^*3*^), respectively. Error bars: SEM. n.s., not significant. **, *P* < 0.01; ***, *P* < 0.005 by Student’s *t*-test. (B, D, F) Comparisons of the geotactic positioning of the flies bearing the *GAL4* transgenes only (*cry-GAL4*/+, *pdf-GAL4*/+ and *pyx-GAL4*/+) and the *UAS-RNAi* transgene only (*UAS-cry RNAi*/+, *UAS-pdf RNAi*/+ and *UAS-pyx RNAi*/+), and the flies with the knockdown of *cry*, *pdf* and *pyx* transcripts (*cry-GAL4*/*UAS*-*cry RNAi*, *pdf-GAL4*/*UAS*-*pdf RNAi* and *pyx-GAL4*/*UAS*-*pyx RNAi*), respectively. Error bars: SEM. n.s., not significant. *, *P* < 0.05; **, *P* < 0.01; ***, *P* < 0.005 by Student’s *t*-test. For all the data, *n* = 10 trials. (PDF 19 kb) Additional file 8: Figure S4.
*cry-, pdf-* and *pyx-GAL4* expressing neurons are necessary for the GMF-induced geotactic positioning under the negative geotactic GMF condition. (A) The geotactic positioning of wing-injured flies. Wings were cut off under CO_2_ anesthesia 24 h before the tube-positioning assay. Anesthetized flies without wing injury were used as controls. (B, C, D) Comparisons of the geotactic positioning of the flies with inhibited CRY-, PDF- and Pyx-expressing neurons by the expression of TNT under the control of *cry-GAL4*, *pdf-GAL4* and *pyx-GAL4*, respectively, under the negative geotactic GMF condition. Error bars: SEM. n.s., not significant. **, *P* < 0.01; ***, *P* < 0.005; ****, *P* < 0.001 by Student’s *t*-test. For all the data, *n* = 10 trials. (PDF 13 kb) Additional file 9: Figure S5. CRY and Pyx function in JO for the near-zero GMF-induced negative geotaxis. (A, C, E) Comparisons of the geotactic positioning of CRY-, Pyx-, and PDF-deficient flies in which the expression of CRY, Pyx, and PDF was genetically restored in JO using *nan-GAL4*, a JO-specific *GAL4* driver. Error bars: SEM. n.s., not significant. *, *P* < 0.05; **, *P* < 0.01; ***, *P* < 0.005, ****, *P* < 0.001 by Student’s *t*-test. (B, D, F) Comparisons of the geotactic positioning of CRY-, Pyx-, and PDF-deficient flies in which the expression of CRY, Pyx, and PDF was genetically restored using another JO-specific *GAL4* driver, *iav-GAL4*. Error bars: SEM. n.s., not significant. **, *P* < 0.01; ***, *P* < 0.005 by Student’s *t*-test. (G, H) Comparisons of the geotactic positioning by wild-type flies, control flies (*GAL4* driver alone, *UAS-pdf RNAi* alone), and the flies with RNAi knockdown of *pdf* using *cry*-*GAL4* or *pyx-GAL4* driver. Error bars: SEM. n.s.: not significant. **, *P* < 0.01; ***, *P* < 0.005; ****, *P* < 0.001 by Student’s *t*-test. For all the data, *n* = 10 trials. (PDF 122 kb)

## Abbreviations

ANOVA: one-way analysis of variance
*cry*: *cryptochrome*
EMF: electromagnetic field
GFP: green fluorescent protein
GMF: geomagnetic field
*iav*: *inactive*
JO: Johnston’s organ
l-LN_v_s: large ventral lateral neurons
LN_d_s: dorsal lateral neurons
*nan*: *nanchung*
*pdf*: *pigment-dispersing factor*
*pdfr*: *pdf receptor*
*pyx*: *pyrexia*
RPM: radical pair mechanism
SEM: standard error of the mean
s-LN_v_s: small ventral lateral neurons
TNT: tetanus toxin
TRP: transient receptor potential
ZT: Zeitgeber time
