# Supplementary figures and images for: Positive geotactic behaviors induced by geomagnetic field in Drosophila
Source: Mol Brain. 2016 May 18;9:55. doi: 10.1186/s13041-016-0235-1 (PMC4870802; doi:10.1186/s13041-016-0235-1)

S1 Fig.

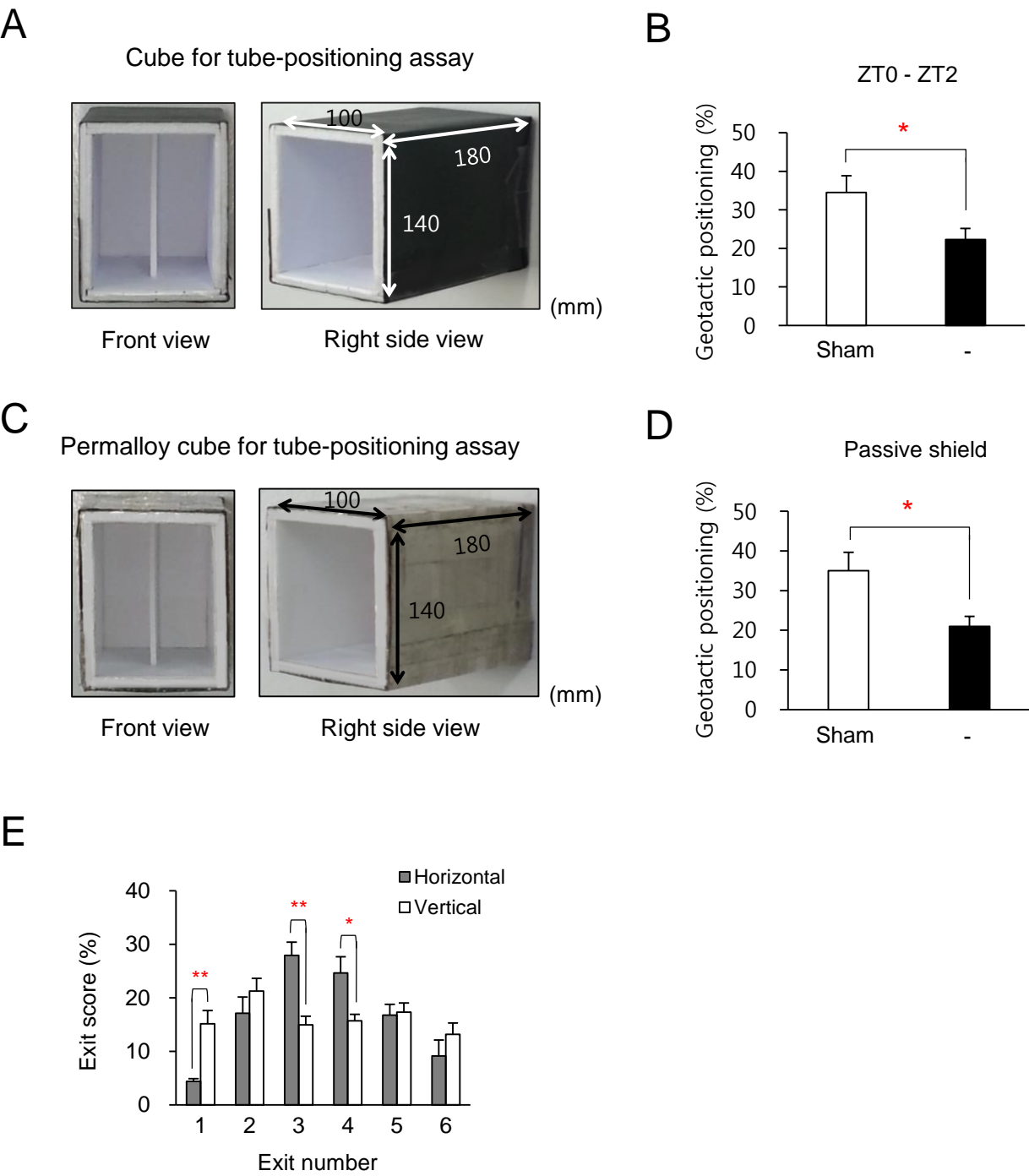

Supplement: Additional file 2: Figure S1. — Near-zero GMF-induced geotactic response under different ZTs and the comparison of vertical choice vs. horizontal choice Y-maze assays. (A) Photos of the test cube (180 × 100 × 140 mm, length × width × height) used for the tube-positioning assay. (B) Comparisons of the geotactic positioning of wild-type flies under the sham and cancellation conditions during ZT0 to ZT2 (n = 10 trials). Error bars: SEM. *, P < 0.05 by Student’s t-test. (C) Photos of the double-layered permalloy (0.5 mm thick) cube (180 × 100 × 140 mm, length × width × height) used for the attenuation of GMF by passive cancellation. (D) Comparisons of the geotactic positioning of wild-type flies under the sham and passive cancellation conditions during ZT5 to ZT8 (n = 10 trials). Error bars: SEM. *, P < 0.05 by Student’s t-test. (E) Comparisons of the exit profiles of wild-type flies making vertical choice versus horizontal choice in the Y-maze assays under the sham condition (n = 12 trials). Error bars: SEM. *, P < 0.05; **, P < 0.01 by Student’s t-test. (PDF 81 kb) [file 13041_2016_235_MOESM2_ESM.pdf]

S2 Fig.

A

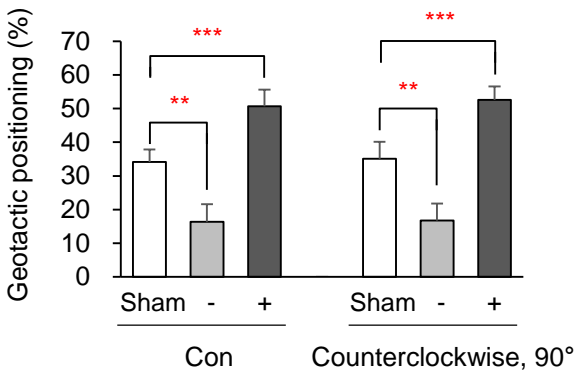

B

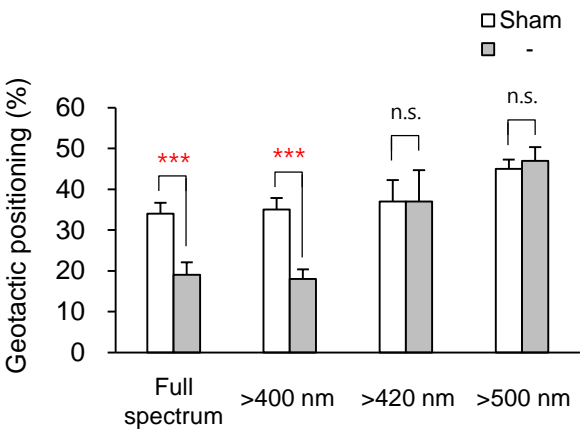

Supplement: Additional file 6: Figure S2. — Comparisons of the geotactic positioning under different direction of the test cube and various light wavelengths. (A) Comparison of geotactic positioning of flies between two different directions, i.e., control and counterclockwise (90°) for the test cube under sham, the shield (−), and the positive geotactic GMF (+). −; near-zero GMF, +; GMF condition b. Error bars: SEM. **, P < 0.01; ***, P < 0.005 by ANOVA Tukey’s test (n = 10 trials). (B) The light intensity was 500 lx in all the experimental conditions. Error bars: SEM. n.s.: not significant. ***, P < 0.005 by Student’s t-test. (n = 10 trials). (PDF 23 kb) [file 13041_2016_235_MOESM6_ESM.pdf]

S3 Fig.

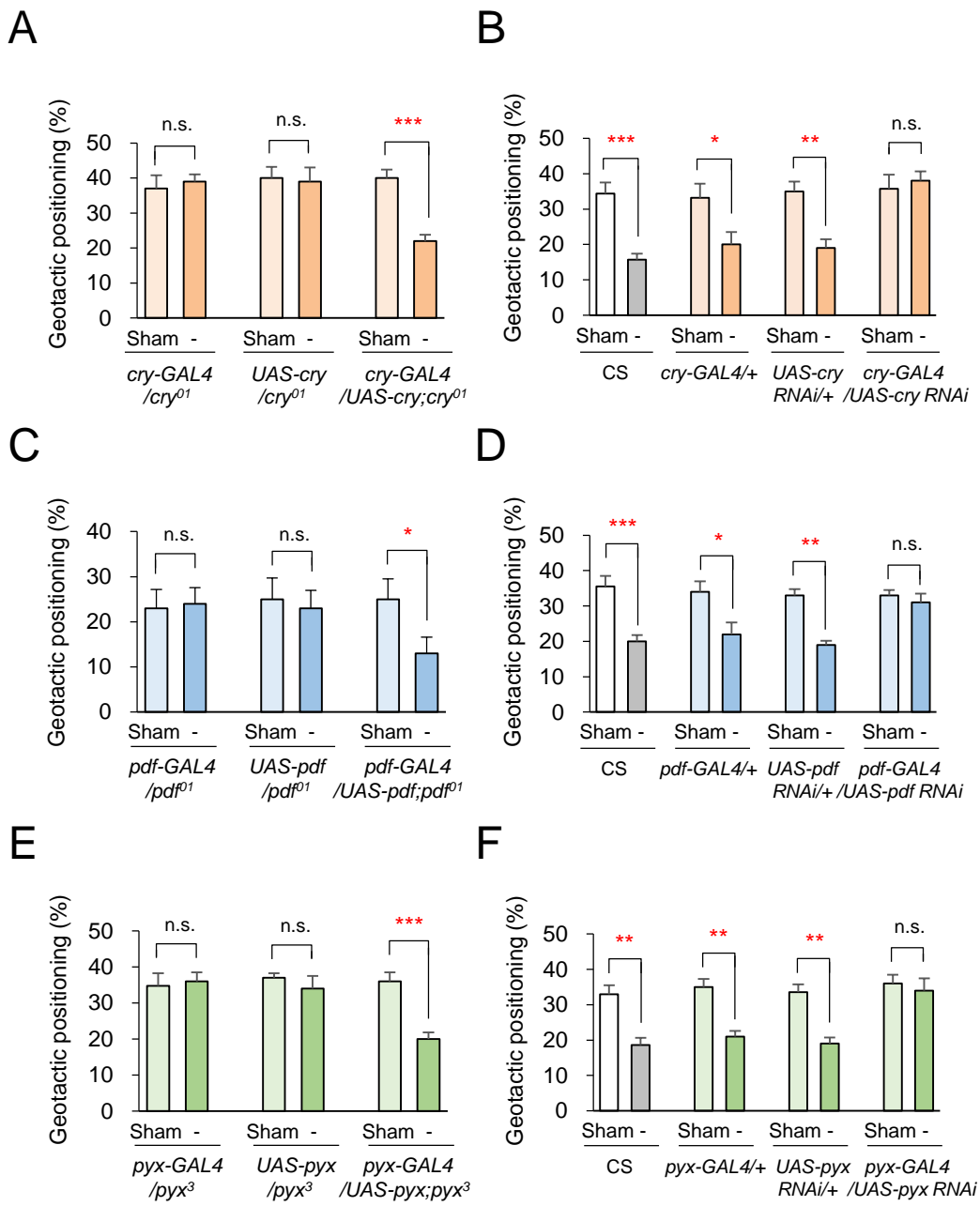

Supplement: Additional file 7: Figure S3. — CRY, PDF and Pyx pathways are required for the near-zero GMF-induced negative geotaxis. (A, C, E) Comparisons of the geotactic positioning of the CRY-, PDF- and Pyx-deficient flies rescued with their coding genes (cry-GAL4/UAS-cry;cry 01, pdf-GAL4/UAS-pdf;pdf 01 and pyx-GAL4/UAS-pyx;pyx 3), respectively. Error bars: SEM. n.s., not significant. **, P < 0.01; ***, P < 0.005 by Student’s t-test. (B, D, F) Comparisons of the geotactic positioning of the flies bearing the GAL4 transgenes only (cry-GAL4/+, pdf-GAL4/+ and pyx-GAL4/+) and the UAS-RNAi transgene only (UAS-cry RNAi/+, UAS-pdf RNAi/+ and UAS-pyx RNAi/+), and the flies with the knockdown of cry, pdf and pyx transcripts (cry-GAL4/UAS-cry RNAi, pdf-GAL4/UAS-pdf RNAi and pyx-GAL4/UAS-pyx RNAi), respectively. Error bars: SEM. n.s., not significant. *, P < 0.05; **, P < 0.01; ***, P < 0.005 by Student’s t-test. For all the data, n = 10 trials. (PDF 19 kb) [file 13041_2016_235_MOESM7_ESM.pdf]

S4 Fig.

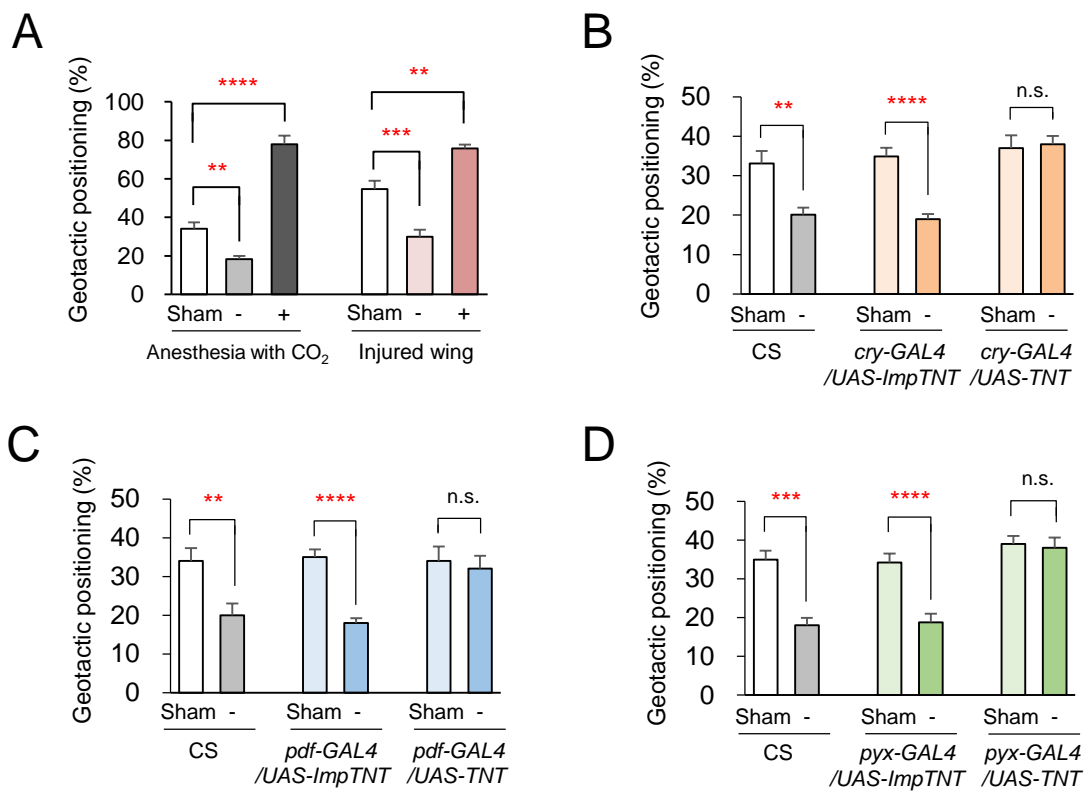

Supplement: Additional file 8: Figure S4. — cry-, pdf- and pyx-GAL4 expressing neurons are necessary for the GMF-induced geotactic positioning under the negative geotactic GMF condition. (A) The geotactic positioning of wing-injured flies. Wings were cut off under CO2 anesthesia 24 h before the tube-positioning assay. Anesthetized flies without wing injury were used as controls. (B, C, D) Comparisons of the geotactic positioning of the flies with inhibited CRY-, PDF- and Pyx-expressing neurons by the expression of TNT under the control of cry-GAL4, pdf-GAL4 and pyx-GAL4, respectively, under the negative geotactic GMF condition. Error bars: SEM. n.s., not significant. **, P < 0.01; ***, P < 0.005; ****, P < 0.001 by Student’s t-test. For all the data, n = 10 trials. (PDF 13 kb) [file 13041_2016_235_MOESM8_ESM.pdf]

S5 Fig.

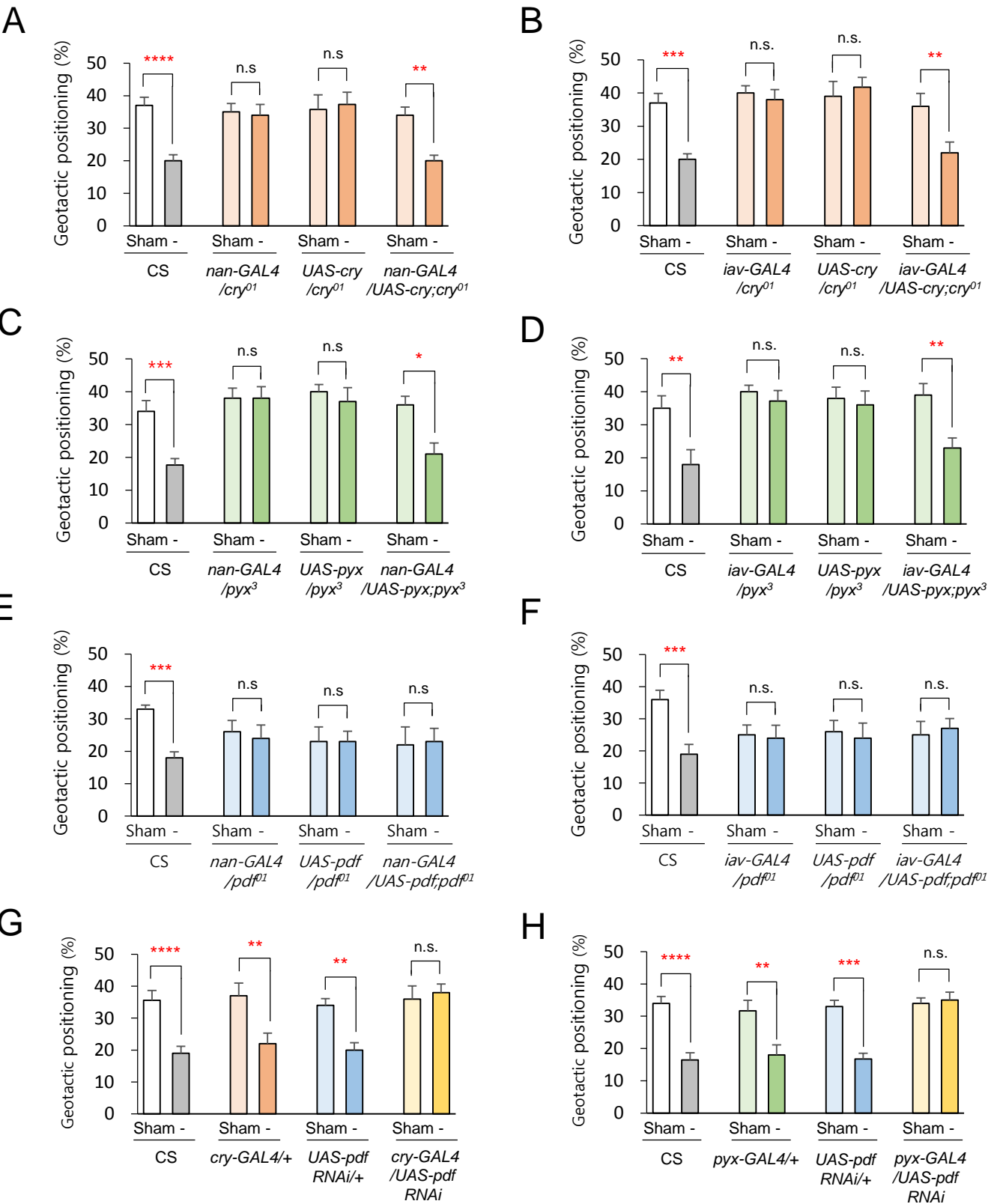

Supplement: Additional file 9: Figure S5. — CRY and Pyx function in JO for the near-zero GMF-induced negative geotaxis. (A, C, E) Comparisons of the geotactic positioning of CRY-, Pyx-, and PDF-deficient flies in which the expression of CRY, Pyx, and PDF was genetically restored in JO using nan-GAL4, a JO-specific GAL4 driver. Error bars: SEM. n.s., not significant. *, P < 0.05; **, P < 0.01; ***, P < 0.005, ****, P < 0.001 by Student’s t-test. (B, D, F) Comparisons of the geotactic positioning of CRY-, Pyx-, and PDF-deficient flies in which the expression of CRY, Pyx, and PDF was genetically restored using another JO-specific GAL4 driver, iav-GAL4. Error bars: SEM. n.s., not significant. **, P < 0.01; ***, P < 0.005 by Student’s t-test. (G, H) Comparisons of the geotactic positioning by wild-type flies, control flies (GAL4 driver alone, UAS-pdf RNAi alone), and the flies with RNAi knockdown of pdf using cry-GAL4 or pyx-GAL4 driver. Error bars: SEM. n.s.: not significant. **, P < 0.01; ***, P < 0.005; ****, P < 0.001 by Student’s t-test. For all the data, n = 10 trials. (PDF 122 kb) [file 13041_2016_235_MOESM9_ESM.pdf]
