# Supplementary material for: Positive geotactic behaviors induced by geomagnetic field in Drosophila
Source: Mol Brain. 2016 May 18;9:55. doi: 10.1186/s13041-016-0235-1 (PMC4870802; doi:10.1186/s13041-016-0235-1)
Supplement: Additional file 5: Table S2. — GMF parameters of the positive geotactic GMF conditions in Fig. 2a. Values of X, Y, and Z intensity are the means of 10 trials for each condition. Total intensity, was calculated using the formula $\sqrt{X^2+{Y}^2+{Z}^2}$X2+Y2+Z2 [13]. a, b, and c are the GMF conditions under which the geotactic positioning was measured, respectively. Note that b and c were positive geotactic GMF conditions. (DOC 28 kb) [file 13041_2016_235_MOESM5_ESM.doc]

| GMF parameter | Sham | *a* | *b* | *c* |
| --- | --- | --- | --- | --- |
| *X* (μT) | 32.24 | 40.00 | 50.00 | 60.00 |
| *Y* (μT) | –5.79 | –5.79 | –5.79 | –5.79 |
| *Z* (μT) | 30.75 | 40.00 | 50.00 | 60.00 |
| Total intensity (μT) | 44.92 | 56.86 | 70.95 | 85.05 |
